# Epigenetic Control in Plants

**DOI:** 10.3390/epigenomes4030011

**Published:** 2020-07-01

**Authors:** Vladimir Brukhin

**Affiliations:** 1 Theodosius Dobzhansky Center for Genome Bioinformatics, St. Petersburg State University, 41 Sredniy Prospekt, Vasilievsky Island, St. Petersburg 199004, Russia; vbrukhin@gmail.com; 2 Department of Plant Embryology & Reproductive Biology, Komarov Botanical Institute RAS, 2 Proffesor Popov Street, St. Petersburg 197376, Russia

Epigenetic regulation in plants is an exciting field of research. It studies the mechanisms and meaning of the phenomena of how a single genome can produce many so-called “epigenomes”. One of the unique property of higher plants that distinguish them from animals is the presence of cells with lifelong totipotency within the meristems. These cells give plants an advantage to reproduce vegetatively and, in fact, they provide a natural possibility of endless cloning of valuable genotypes. During plant development, epigenetic mechanisms gradually restrict cell totipotency, providing cell specialization and, ultimately, the formation of plant tissues and organs. In addition, largely due to epigenetic rearrangements, plants change the expression of their genes in response to changing environmental conditions, which is especially important, given that most plants themselves cannot move far away. Finally, epigenetic regulation is indispensable for the response and adaptation of plants to the emerging stresses that they experience at times. The investigation of the reasons why and how one plant genotype can produce many phenotypes and where the point of no return in cell type specialization occurs is extremely important for plant developmental biology and genetics. 

In the second half of the twentieth century, after the introduction of the term “epigenetics” by Conrad Hal Waddington, much has been done in this field by the efforts of so-called "wet biologists" (experimental biologists working in labs). It was found that the main mechanisms of epigenetic regulation in eukaryotes operate via DNA methylation, chromatin remodeling, and RNA interference. It was found that epimutations are often precursors of the mutations per se, that epigenetic events are involved in, gene suppression and co-suppression, inactivation of transposons, genetic imprinting, transgene silencing, etc. In the last two decades, with the advent and rapid progress in next-generation sequencing, computational biology (dry biology), and machine learning methods, the study of epigenomes has become possible not only at a whole plant genome level, but also at the level of plant communities and populations of epigenomes. This suggests anticipation of new intriguing discoveries in plant epigenomics at a fairly rapid rate and on a larger scale compared to the previous decades.

